# Exploration of the typical features of tubulovillous adenoma using in-depth quantitative proteomics analysis

**DOI:** 10.1080/21655979.2021.1971036

**Published:** 2021-09-29

**Authors:** Yin Zhang, Meng Pan, Chun-Yuan Li, Jing-ying Li, Wei Ge, Lai Xu, Yi Xiao

**Affiliations:** a Department of General Surgery, Division of Colorectal Surgery, Peking Union Medical College Hospital, Chinese Academy of Medical Sciences and Peking Union Medical College, Beijing, China; b State Key Laboratory of Medical Molecular Biology & Department of Immunology, Institute of Basic Medical Sciences Chinese Academy of Medical Sciences, School of Basic Medicine Peking Union Medical College, Beijing, China

**Keywords:** Tubulovillous adenoma, tumorigenesis, PLOD3, quantitative proteomics, metabolic reprogramming, tumor microenvironment

## Abstract

This present study aimed to explore the typical protein features of tubulovillous adenoma (TVA) using proteomic and bioinformatic analyses. Tandem mass tag (TMT)-based quantitative proteomic analyses were conducted on normal mucosa, tubular adenoma, TVA and adenocarcinoma tissues. We identified 5,665 proteins categorized into seven clusters based on Pearson’s correlation analysis. The bioinfomatic analysis showed mitochondrial and metabolism-related events were typical characteristics of TVA and mitochondrial-, ribosome- and matrisome-related biological processes may contribute to carcinogenesis. PLOD3 was identified as a key protein associated with the malignant potential of TVA and promoted the viability of adenoma organoids. The Cancer Genome Atlas (TCGA) analysis revealed PLOD3 as a risk factor for disease-free and overall survival. Furthermore, the PLOD3 expression correlated negatively with the abundance of B cells, CD8 + T cells, CD4 + T cells, neutrophils, macrophages and myeloid dendritic cells. In conclusion, enhanced metabolic and mitochondrial reprogramming are typical features of TVA, and PLOD3 might be related to the “immune desert” phenotype and contribute to TVA tumorigenesis and colorectal cancer development.

## Introduction

Colorectal cancer (CRC) is the third most commonly diagnosed cancer in the world and the second leading cause of cancer deaths [1]. A comprehensive understanding of the evolution of the CRC is of critical importance in improving screening and prevention. In the canonical carcinogenesis of CRC, the conventional adenoma-to-carcinoma (A-C) sequence accounts for approximately 85% of the cases [2]. However, only 5% of the conventional adenomas could finally become carcinoma [3]. Therefore, classification of conventional adenoma based on the risk of carcinogenesis is essential for accurate surveillance. Patients with advanced adenoma (tumor size >1 cm, villous component (VC) >25% or/and high-grade intraepithelial neoplasia) have a higher risk of developing cancer and cancer mortality compared with non-adenoma patients, while the patients with non-advanced adenoma do not [4].

At the histological level, adenoma with VC is the representative type of advanced adenoma. According to the proportion of the VC, VC-type is categorized as tubulovillous adenoma (TVA, 25%–75% VC) and villous adenoma (VA, >75% VC) [5]. Using tubular adenoma (TA) as a reference, the relative risks of malignant transition of TVA and VA are 1.51 and 2.53, respectively, with a higher proportion of VC correlated with a higher risk of malignant transition [6]. Hence, it is important to determine the features that characterize the high-risk malignant transition of TVA/VA. At the genetic level, VC is significantly associated with *Kras* mutations, but without correlation to adenoma size or the number of chromosomal abnormalities [3]. Activating *GNAS* mutations occur frequently in VA, but these mutations are irrelevant to carcinogenesis of VA [7,8]. At the epigenetic level, the CpG island methylator phenotype-positive status is frequently identified in TVA/VA and the degree of MGMT and RASSF2 methylation is significantly higher in TVA/VA than in TA [9]. At the gene expression level, AMN and PTGDR were found to be downregulated, while osteopontin and osteonectin were upregulated in the VA-carcinoma sequence [10].

In this study, we aimed to provide a comprehensive description of the typical protein features of TVA and identify key proteins that reflect the potential malignant risk of TVA with proteomic strategy and bioinformatic analysis. Our results could contribute to the further understanding of the features and carcinogenesis potential of TVA at the protein level.

## Methods

### Patients and tissue samples

Normal mucosa (NM), TA, TVA and AC tissues were obtained from 24 patients recruited at the Division of Colorectal Surgery, Department of General Surgery, Peking Union Medical College Hospital (China). Details of the patients and tissues are listed in Table S1. After removal, all tissues were stored temporarily on dry ice and then transferred to −80°C.

This study was approved by the Ethics Committee of Peking Union Medical College Hospital (Number: JS-2094). Written informed consent was obtained from each patient prior to the study commencement.

### Protein extraction and tandem mass tag-labeling (TMT-labeling)

Lysate proteins (100 µg) in 8 M urea were alkylated with dithiothreitol (DTT) and iodoacetamide (IAA) before protein digestion. The proteins were digested with Trypsin/Lys-C and protein labeling was performed using the TMT-labeling kit according to the manufacturer’s instructions. The TMT-labeled peptides were analyzed by high performance liquid chromatography with a directly interfaced Thermo Orbitrap Fusion mass spectrometer (Thermo Scientific, USA). Protein identification was performed using Proteome Discoverer 2.2 software (Thermo Scientific, USA) with the SEQUEST search engine. Proteins with a false discovery rate (FDR) <0.01 and unique peptides ≥2 qualified for further analysis. Relative protein quantification was performed using the TMT-6plex method. All the methods were performed as previously described [11]. The mass spectrometry proteomics data have been deposited with the ProteomeXchange Consortium (http://proteomecentral.proteomexchange.org) in the iProX partner repository with the dataset identifier: PXD023899 [12].

### Bioinformatic analysis

Heatmaps were generated using the HCE 3.5 software based on the standardized proteomic profilings of NM, TVA and AC [13]. Gene ontology (GO), Kyoto Encyclopedia of Genes and Genomes (KEGG) and Wiki-pathway analyses were performed using the ClueGO and CluePedia plugins in Cytoscape [14–16]. Results with *P <* 0.05 and validated experimentally could be visualized. Protein–protein interaction (PPI) networks were constructed using the Search Tool for the Retrieval of Interacting Genes (STRING) database [17]. The annotation and biological process categories were downloaded in Metascape database [18]. Survival analysis was performed using the Gene Expression Profiling Interactive Analysis (GEPIA) database [19]. The Cancer Genome Atlas (TCGA) data of CRC was downloaded using cBioPortal database [20,21]. Immune cell infiltration was estimated using the RNA-seq data using the TIMER database [22].

### Generation of adenoma organoids

For human adenoma organoids, fresh adenoma tissue was cut into small pieces, washed with PBS and dissociated in TrypLE (Gibco, 12,604–013) to achieve cell suspension. The culture medium was prepared using Sato’s method [23]. The organoids were passaged (1:3) every week. Appropriate Cell Recovery Solution (Corning, REF354253) was added to the Matrigel and dissociated for 1 h at 4°C to obtain the organoid suspension. Then the supernatant was discarded after centrifugation, the organoids were embedded in the Matrigel.

For mouse adenoma organoids, the intestinal fragments containing adenomas from C57BL/6 J-*APC^Min^* mice (male, 16 weeks old, Model Animal Resource Information Platform) were treated as described for the human adenoma tissue. The culture medium was prepared as follows: penicillin 1 U/mL, streptomycin 1 μg/mL, HEPES 10 mmol/L, Glutamax, 1x N2, 1x B27 and N-acetylcysteine 1 mmol/L in Advanced Dulbecco’s Modified Eagle’s Medium and F12.

### RNA extraction and qRT-PCR analysis

Total RNA was extracted from HCT116 cells and adenoma organoids using TRIzol reagent (Thermo Scientific, 155,996,018) according to manufacturer’s instructions. We used HCT116 cells to detect the knockdown efficiency of PLOD3. RNA concentrations of cells and adenoma organoids were measured by NanoDrop spectrophotometer (Thermo Scientific). Real-time quantitative PCR was performed using the SYBR-Green (Takara, RR096A), and the relative PLOD3 mRNA expression levels were normalized to that of GAPDH. Primers used in this experiment were as follows:

GAPDH (forward: 5ʹ-GTCTCCTCTGACTTCAACAGCG-3ʹ,

reverse: 5ʹ-ACCACCCTG-TTGCTGTAGCCAA-3ʹ);

PLOD3 (forward: 5ʹ-CTGAAGAAGTTCGTCCAGAGTG-3ʹ,

reverse: 5ʹ- ACCGATGAATCCACCAGAATTG-3ʹ).

### Lentiviral transduction of adenoma organoids

The organoids were harvested and dissociated in TryplE for 5 min in a 37°C incubator. After terminating digestion, the organoids were centrifuged to pellet for 5 min at 500 × g. Then the organoids were resuspended in 500 μL lentiviral transduction medium and transferred into a 12-well culture plate. The plate was centrifuged at 600 × g at 32°C for 1 h and placed in the 37°C incubator for another 4 h. Transferred the organoid-virus mixture into a tube and centrifuged to discard the supernatant. The pellet was resuspended in Matrigel and cultured for 48 h at 37°C.

### Immunofluorescence

Organoids were isolated from the Matrigel and then fixed with 4% paraformaldehyde for 1 h at room temperature. After three washes with PBS, the organoids were blocked and permeabilized with PBS containing 5% goat serum, 0.5% Triton X-100 and 0.5% NaN_3_ for 2 h at room temperature. And then, the organoids were incubated with primary antibodies Ki67 (abcam, ab15580, 1:50) and PCNA (Origene, TA800875, 1:50) separately. After that, the organoids were incubated with Alexa Fluor 488 (Thermo, A-11034, 1:50) and Alexa Fluor 594 (Thermo, A-11032, 1:50) for 2 h at room temperature. Next, the organoids were stained with 5 μg/mL DAPI (Solarbio, C0065) for 15 mins at room temperature. After washing with PBS, the images were captured with a confocal microscope (Zeiss LSM 780).

### 3D cell viability assay

The adenoma organoids transfected with PLOD3-siRNA were used to detect cell viability. The CellTiter-Glo® 3D Reagent (Promega, G9681) was used to dissolve the matrigel. The adenoma organoid suspension was equally distributed to opaque-walled multiwell plates (Corning, REF3603). The volume of CellTiter-Glo® 3D Reagent equal to the volume of cell culture medium was added to each well and then the content was incubated for 30 min at room temperature. Cell viability was calculated based on ATP content of each well.

### Statistical analysis

Statistical analysis was performed using GraphPad Prism 8.0.1 (GraphPad Software, Inc., La Jolla, CA, USA). Quantitative data were analyzed using Student’s *t*-test or ANOVA. Pearson’s correlation analysis was performed to evaluate associations between sets of data. The Kaplan–Meier method was used for survival analysis. *P* < 0.05 was considered to indicate statistical significance.

## Results

In the present study, we aimed to explore the typical protein features of TVA by proteomic strategy and bioinformatic analysis. We found that the typical protein features of TVA are mainly enriched in metabolism related biological process and pathway. Then we identified PLOD3 as the potential malignant predictive protein of TVA and validated the function of PLOD3 on adenoma organoids. Furthermore, we find that PLOD3 might be related to the immune desert phenotype of CRC and be a risk factor of CRC.

### Screening for the protein clusters representative of TVA

**Figure 1.**
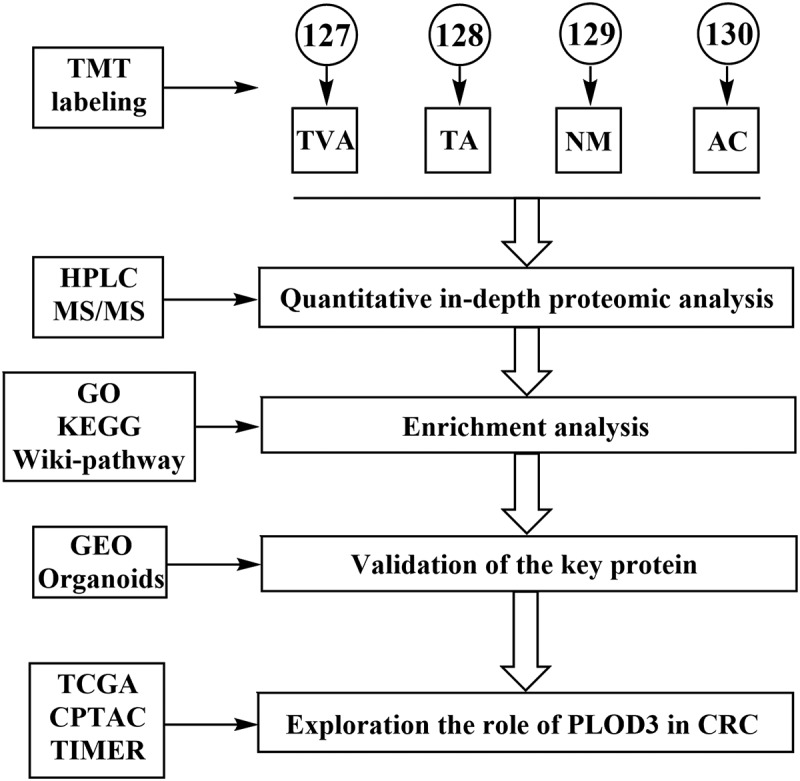
Workflow of the study

**Figure 2.**
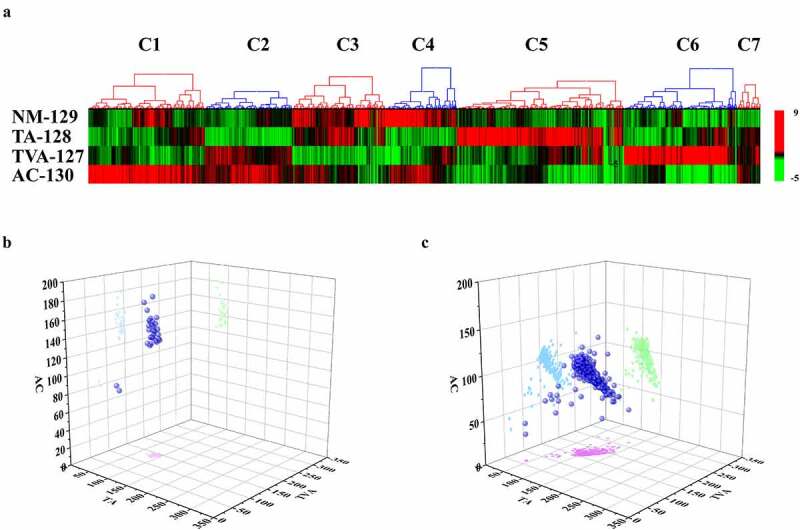
Identification of the highly expressed proteins in tubulovillous adenoma

The clinical characteristics of the NM, TA, TVA and AC tissue samples obtained from patients are listed in Table S1. The workflow of this study is shown in Figure 1. A total of 5,665 proteins were identified according to the criteria described in the Methods section. The proteins were classified into seven clusters as shown in Figure 2a. This heatmap revealed that the proteins in the cluster 6 were highly expressed in TVA (950 proteins) and the proteins in cluster 2 were highly expressed in both TVA and AC (734 proteins). Based on the theory that TVA is more liable to evolve into cancer than TA, we speculated that the proteins in cluster 2 might contribute to the carcinogenesis because of the similarity in the expression pattern between TVA and AC. The proteins in clusters 2 and 6 were regarded as the representative clusters of TVA and selected for further analysis.

A. Heat map showing the relative abundance of 5,665 proteins in the normal mucosa (NM), tubular adenoma (TA), tubulovillous adenoma (TVA) and Adenocarcinoma (AC) group. The proteins were clustered hierarchically by Pearson correlation analysis. Green indicates downregulated proteins, whereas red indicates upregulated proteins. B-C. Figures 2b and 2c are three-dimensional diagrams of the expression patterns of the filtered proteins in clusters 2 and cluster 6. The filtered proteins of cluster 2 are the highly expressed proteins related to both TVA and AC. The filtered proteins of cluster 6 are the highly expressed proteins related to TVA only. Three-dimensional space was constructed with protein expression value of TVA, AC and TA tissues as the coordinate axis. The expression level of the filtered cluster 2 and cluster 6 proteins in TVA, AC and TA tissues are indicated by blue balls. The light blue dots are the projection of the protein on the AC and TVA coordinate axes, showing the expression level of the proteins in AC and TVA tissues. Similarly, the pink dots indicate the expression level of the protein in TA and TVA tissues, and the green dots indicate the expression level of the protein in TA and AC tissues. All the protein names in Figures 2b and 2c were listed in Supplementary file 1.

### Identification of the typical features of TVA

**Figure 3.**
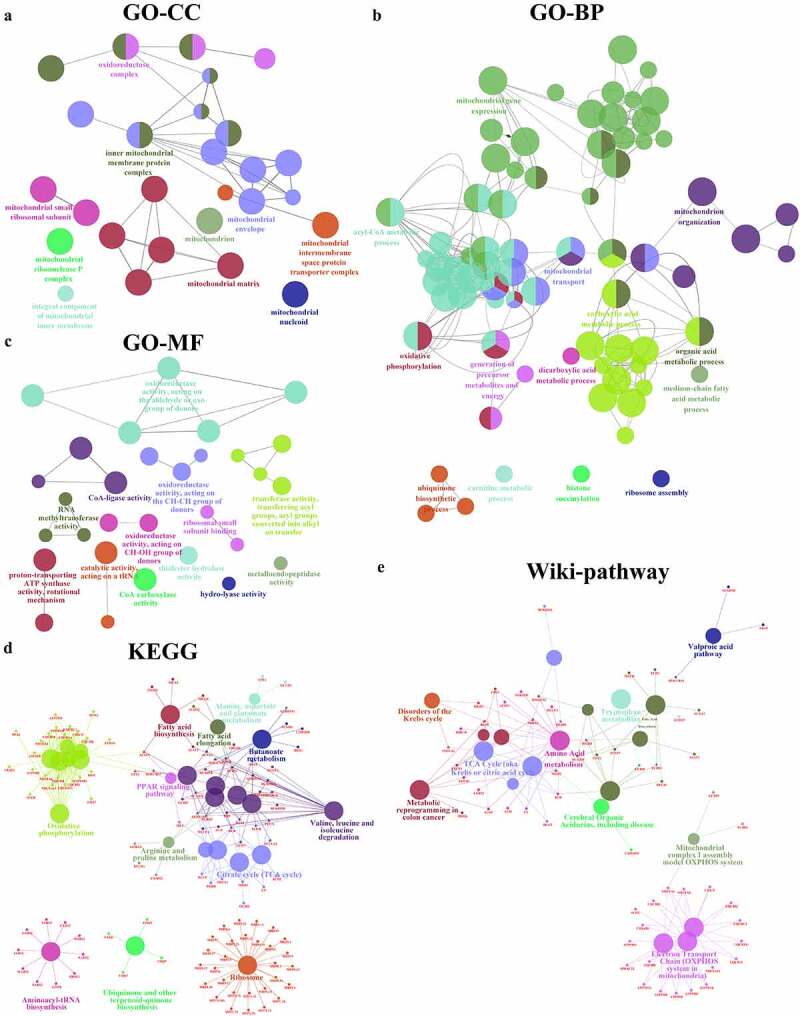
Enrichment analysis of the highly expressed proteins in cluster 6

**Figure 4.**
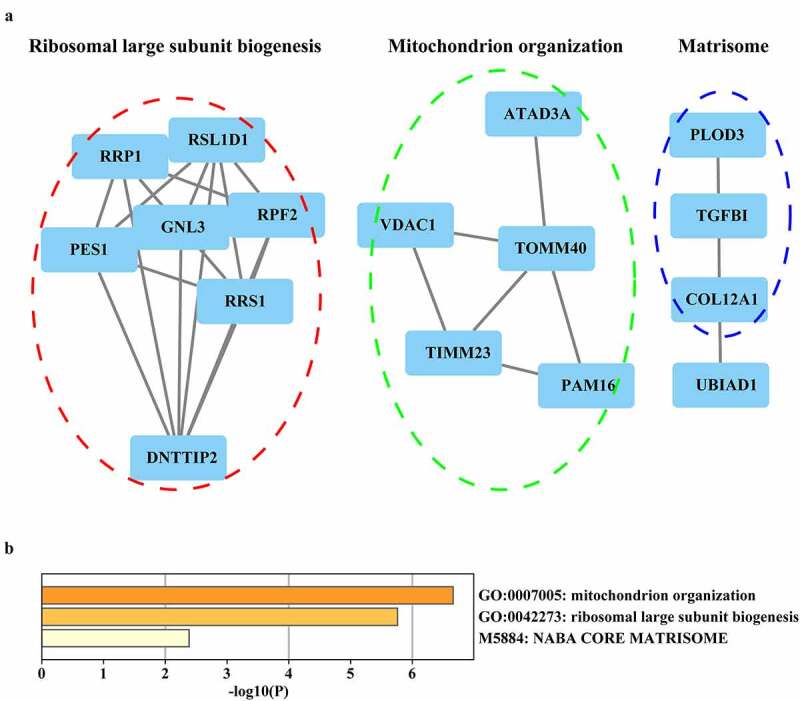
Enrichment analysis of the highly expressed proteins in cluster 2. (A) Protein–protein interaction (PPI) network of the highly expressed proteins in cluster 2. (B) GO-BP and pathway analysis of the highly expressed proteins in cluster 2

To identify the biological processes and pathways typical of TVA, we selected the highly expressed proteins related to TVA in cluster 6 with a fold-change (FC) in expression >1.5 in 127/128, 127/129 and 127/130 (265 proteins, Supplementary file 1). We selected the highly expressed proteins related to both TVA and AC in cluster 2 following the FC >1.5 in 127/128, 127/129, 130/129 and 130/128 (33 proteins, Supplementary file 1). The expression profile of the highly expressed proteins in clusters 6 and 2 are shown in Figure 2b-c. We performed Gene Ontology (GO) analysis of the highly expressed proteins in three aspects: GO-cellular component (CC), GO-molecular function (MF) and GO-biological process (BP). The aim of GO analysis was to investigate the main location of the cell, activities at the molecular level and biological phenomenon of the highly expressed proteins in cluster 6. GO-CC enrichment analysis showed that most of the proteins were located in the mitochondria (Figure 3a). Mitochondrial gene expression, acyl-CoA metabolic process and carboxylic acid metabolic process were mainly enriched in GO-BP analysis (Figure 3b). Oxidoreductase activity and CoA-ligase activity were mainly enriched in the GO-MF analysis (Figure 3c). We performed a KEGG pathway analysis to investigate which pathway the proteins were mainly involved in. The result showed that valine, leucine and isoleucine degradation, citrate cycle, oxidative phosphorylation and mitochondrial ribosome-related pathway were mainly enriched in the KEGG pathway analysis (Figure 3d). The results of GO and KEGG analysis indicated that the formation of the villous component is related to enhanced metabolic activities. To further validate and supplement the results of GO and KEGG analysis, we performed Wiki-pathway analysis. The advantage of Wiki-pathway analysis is that Wiki-database supports crowdsourcing and is updated in a timely manner. Furthermore, Wiki-pathway analysis shows favorable interactivity in metabolic pathways [24]. The enrichment result of Wiki-pathway is similar to the KEGG analysis. The proteins were mainly enriched in metabolism-related pathway. Moreover, Wiki-pathway analysis showed that the proteins were related to metabolic reprogramming in colon cancer (Figure 3e). Metabolic reprogramming is one of the hallmarks of cancer [25] . This result indicates that the metabolic reprogramming events in CRC might initiate in the TVA phase. The construction of the PPI network of the highly expressed proteins in cluster 2 showed that mitochondrial-, ribosome- and matrisome-related biological processes and pathways were enriched (Figure 4a-b). Matrisome-related pathway was the representative in cluster 2 and PLOD3, TGFBI and COL12A1 were the annotation proteins in this pathway. Moreover, PLOD3 and COL12A1 were related to the epithelial–mesenchymal transition (EMT) pathway, which is regarded as one of the core events that contribute to cancer metastasis. Therefore, we speculated that the matrisome-related pathway is associated with a higher potential for carcinogenesis of TVA compared to TA and we focused on PLOD3 and COL12A1 for further analyses.

Gene ontology (GO), Kyoto Encyclopedia of Gene (KEGG) and Wiki-pathway analyses of the highly expressed proteins in cluster 6 were performed using the ClueGO and CluePedia plugins in Cytoscape. Circles shown in the same color represent similar enrichment results. (A) GO-cell component (CC) analysis of the highly expressed proteins in cluster 6. (B) GO-molecular function (MF) analysis of the highly expressed proteins in cluster 6. (C) GO-biological process (BP) analysis of the highly expressed proteins in cluster 6. (D) The KEGG analysis of cluster 6 (E) Wiki-pathway analysis of cluster 6.

### PLOD3 promotes the viability and proliferation of adenoma organoids

**Figure 5.**
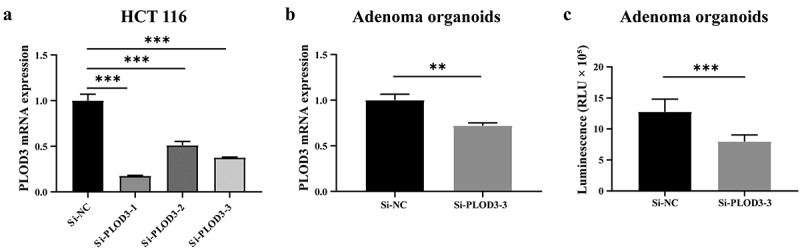
PLOD3 knockdown reduces viability of adenoma organoids. (A) Expression level of PLOD3 mRNA in HCT116 cells was evaluated by qPCR after knockdown mediated by three si-RNA sequences. (B) Expression level of PLOD3 mRNA in adenoma organoids was significantly decreased after knockdown mediated by the si-3 RNA sequence. (C) The viability of adenoma organoids was significantly decreased after PLOD3 knockdown

To further explore the key proteins that may contribute to the carcinogenesis of TVA, were focused on PLOD3 and COL12A1, which are proteins related to the EMT pathway. PLOD3 is a multifunctional protein that promotes collagen maturation by catalyzing hydroxylation and glycosylation of lysine residues in procollagen, while COL12A1 is a collagen protein that is a component of the extracellular matrix. Comparison of the expression of PLOD3 and COL12A1 in TVA and TA using the Gene Expression Omnibus database (GSE 117607) showed higher specificity of PLOD3 in TVA (Figure S1). Therefore, based on its multifunctional characteristics and higher specificity in TVA, we selected PLOD3 for further analysis. After confirming the knockdown ability of si-RNA-mediated knockdown of PLOD3 in adenoma organoids generated from fresh surgical specimens, we investigated its effects on viability. The viability of si-PLOD3 organoids was reduced compared with that of control organoids (Figure 5a-c). Then we explored the overexpression effect of PLOD3. Compared with the control group, adenoma organoids showed increased cell viability and more Ki67- and PCNA-positive cells (Figure S3). The functional experiments indicated that PLOD3 promotes the viability and proliferation of adenoma. Since the acquisition of increased viability and proliferation of premalignant lesions is a pivotal event in carcinogenesis, we speculated that PLOD3 plays a key role in the carcinogenesis of CRC.

### Exploration the role of PLOD3 in CRC

**Figure 6.**
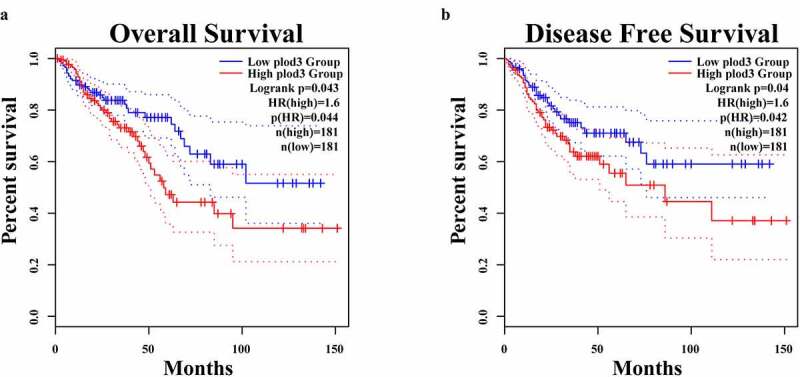
Survival analysis of PLOD3 in CRC patients. (A) The disease-free survival (DFS) of the high PLOD3 expression group is significantly poorer than that of the low PLOD3 expression group in the GEPIA database (*P = *0.04, hazard ratio (HR) = 1.6, 362 patients). (B) The overall survival (OS) of the high PLOD3 expression group is significantly poorer than that of the low PLOD3 expression group in GEPIA database (*P = *0.043, HR = 1.6, 362 patients)

**Figure 7.**
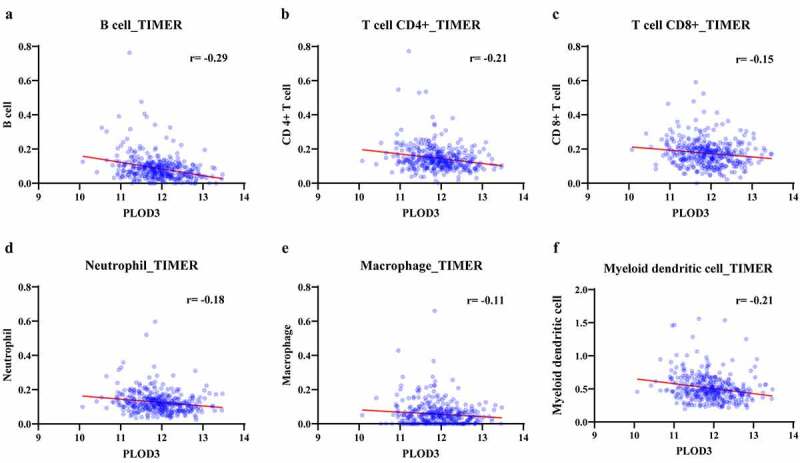
The correlation analysis between PLOD3 expression and immune cells. (A-F) PLOD3 negatively correlated with B cell, CD4 + T cell, CD8 + T cell, neutrophil, macrophage, myeloid dendritic cell infiltration in the TCGA database using TIMER algorithm (*P* < 0.05)

Analysis of the proteins in the cluster 2 indicated that PLOD3 has the potential to promote TVA carcinogenesis. We next evaluated the correlation between PLOD3 and survival, clinical stage and the tumor microenvironment (TME) of CRC based on public databases. GEPIA is an online database that contains the survival data of the CRC patients in TCGA database. The high PLOD3 expression was associated with poor overall survival (OS) and disease-free survival (DFS) in the GEPIA database (Figure 6a-b). Furthermore, PLOD3 expression was significantly higher in the patients with distant metastasis (M1) than in the M0 patients based on TCGA database (Figure S2A). PLOD3 expression was higher in the patients with local lymph node metastasis (N+) than in the N0 patients, although this difference was not statistically significant (Figure S2B). In the CPTAC database, there was no difference in PLOD3 expression between the M0 and M1 patients, although this result may be influenced by the imbalanced sample size (Figure S2C). The expression of PLOD3 was significantly higher in N+ patients than in N0 patients (Figure S2D). We further explored the correlation between PLOD3 and tumor infiltrating immune cells. Based on the analysis of TCGA database using the TIMER method, the PLOD3 expression showed a significantly negative correlation with the abundance of B cells, CD8^+^ T cells, CD4^+^ T cells, neutrophils, macrophages and myeloid dendritic cells (Figure 7).

## Discussion

In this study, we performed quantitative proteomics on NM, TA, TVA and AC tissues and conducted a systematic analysis on the proteomic profile of TVA. We found that the typical proteins of TVA were located mainly in the mitochondria and enriched in metabolic events. We then compared the similar proteomic clusters identified in the analysis of TVA and AC samples. We found that matrisome-related proteins were highly expressed in both TVA and AC and these proteins might be relevant to potential malignant features of TVA. PLOD3, an EMT-related protein, was identified as the key protein in the TVA-AC process among the matrisome-related proteins. The function of PLOD3 was validated using adenoma organoids. Moreover, our findings indicated that PLOD3 is associated with local or distant metastasis, poor prognosis and the ‘immune desert’ phenotype in CRC.

Enrichment analysis of the typical proteins of TVA indicated that TVA possesses a high metabolic status compared with TA and AC. Moreover, we found that in the TVA stage metabolic reprogramming events could occur and the main metabolic mode of TVA was oxidative phosphorylation, branched chain amino acids (BCAA) degradation and mitochondrial ribosome-related pathway. TVA/VA has a higher proportion of hyperplastic epithelial cells, which reflects a greater proliferative ability and energy requirement compared with TA [26]. The histological features could partially explain the high metabolic status of TVA. Some studies showed analogous results that enhanced metabolic related pathway and metabolic reprogramming are initiated in the pre-malignant stage of CRC. In one study of the metabolic and mitochondrial reprogramming events in the pre-malignant lesions of the rectal mucosa, Cruz et al. found that the Warburg effect and metabolic reprogramming existed prior to the pre-malignant status and that mitochondrial numbers were increased in both biopsy tissues and animal models [27]. Similarly, Bensard et al. demonstrated that metabolic reprogramming of aerobic glycolysis, a pattern that is known as the Warburg effect and regarded as the hallmark of most cancer cells, promoted tumor initiation in CRC [25,28,29]. Satoh et al. found that metabolic reprogramming occurred at the adenoma stage and metabolism-related genes were regulated by MYC [30]. However, it is worth noting that in our study the main mechanism of energy generation in TVA is the mitochondrial oxidative phosphorylation rather than aerobic glycolysis. We therefore consider that the evolution of CRC is a complex process and the metabolic and mitochondrial reprogramming associated with the NM-TVA-AC sequence represents a fluctuation rather than a monotonic change. It possibly means that oxidative phosphorylation is the main metabolic pathway in the NM-TVA sequence and aerobic glycolysis are enhanced in the TVA-AC sequence. We have reviewed the relevant studies and found that the tumorigenesis process of some types of CRC showed similar metabolism fluctuating mode. A bimodal change in the mitochondrial DNA copy number has been identified in the evolution of ulcerative colitis-related cancer [31]. In one study of familial adenomatous polyposis tumorigenesis, the genes related to the TCA cycle also triggered bimodal changes in expression based on a pseudotime trajectory [32]. In addition to the oxidative phosphorylation pathway, we found that the BCAA degradation and mitochondrial ribosome-related pathways were significantly enriched in TVA. Also, plasma concentrations of BCAAs are negatively correlated to the risk of colorectal adenoma [33]. In the obesity colon cancer model, BCAA supplementation prevented the initiation of colonic pre-neoplastic lesions [34]. Therefore, BCAA intake represents a potential chemo-prevention strategy for CRC. Abnormal expression of mitochondrial ribosome proteins (MRPs) was related to mitochondrial metabolism disorder and cell dysfunction and was observed in a variety of cancer types [35]. MRPL35 was found to be upregulated in CRC and associated with poor survival in a Chinese cohort [36]. The specific role of MRPs in the initiation and development of CRC and the underlying mechanism remains to be fully elucidated.

In addition to exploring the clusters and proteins characteristic of TVA, we identified a cluster of proteins that showed similar expression modes across TVA and AC. These proteins might be associated with the malignant potential of TVA. In constructing the PPI network, we focused on the matrisome-related proteins and selected PLOD3, an EMT-related protein, as the key protein that represents the higher malignant potential of TVA compared with TA. EMT-related proteins were regarded as risk factors for CRC in the previous studies. Huang et al. found that an EMT-related gene expression signature combining infiltrating T-cell abundance has showed promising prognostic value in colon cancer [37]. Further, EMT-related gene expression signature can also be applied as a risk stratified tool in hepatocellular cancer [38]. In our study, PLOD3 plays an important role not only in CRC, but also in the TVA-AC sequence. At the adenoma organoid level, we found that PLOD3 can promote the viability and proliferation of adenoma. Thus, the functional experiments validated the key role of PLOD3 in the tumorigenesis of CRC. Besides the function of PLOD3 at the adenoma level, we also found that PLOD3 is upregulated in tumor tissue. PLOD3 is a multifunctional enzyme with lysyl hydroxylase, collagen galactosyltransferase as well as glucosyltransferase ability and is highly expressed and associated with the progression of lung cancer, glioma, ovarian cancer, gastric cancer, hepatocellular cancer and pancreatic cancer [39–45]. In line with our findings, Nicastri et al. found that PLOD3 was upregulated in CRC tissue using the quantitative proteomic technique focusing on N-linked glycoproteins [46]. In the hepatocellular cancer, PLOD3 was identified as one of the early-stage decision markers that were upregulated in the premalignant lesions [44]. This result further has indicated the potential key role of PLOD3 in other cancer types. In our analysis of the CRC cohort in TCGA, we found that high PLOD3 expression was significantly associated with poor survival of patients, and PLOD3 was negatively correlated with the immune cells infiltrating in TME. This phenomenon conforms to the immune desert type of TME, in which the typical cancer category is Microsatellite stable-CRC (MSS-CRC) initiated from the adenoma-carcinoma sequence, and confirmed the reliability of our data analysis [47]. Therefore, we speculated that the immune cell-excluded events can occur in the pre-cancer stage.

Two limitations of this study are specified as follows. First, since the results are based mainly on proteomic analysis, the potential mechanisms underlying the role of PLOD3 in the tumorigenesis and development of CRC remain to be elucidated. Second, our analysis showed that metabolic and mitochondrial reprogramming of oxidative phosphorylation and the BCAA degradation and mitochondrial ribosome-related pathways occurred in the TVA stage. This phenomenon needs to be further validated in mechanistic and metabolomic studies.

## Conclusion

In conclusion, we comprehensively analyzed the proteomic profiles of NM, TA, TVA and AC. Enhanced metabolic and mitochondrial reprogramming, such as oxidative phosphorylation, BCAA degradation and mitochondrial ribosome-related pathway were identified as typical features of TVA. Furthermore, we showed that PLOD3, which was correlated with an ‘immune desert’ type TME, might contribute to TVA tumorigenesis and CRC development.

## Supplementary Material

Supplemental Material

## Data Availability

The mass spectrometry proteomics data have been deposited to the ProteomeXchange Consortium (http://proteomecentral.proteomexchange.org) via the iProX partner repository with the dataset identifier: PXD023899.
