# Supplementary material for: Exploration of the typical features of tubulovillous adenoma using in-depth quantitative proteomics analysis
Source: Bioengineered. 2021 Sep 29;12(1):6831–43. doi: 10.1080/21655979.2021.1971036 (PMC8806592; doi:10.1080/21655979.2021.1971036)
Supplement: Supplemental Material [file KBIE_A_1971036_SM7445.zip › supplementary/Table S1.docx]

**Table S1. Clinical and pathological characteristics of patients.**

| **Patient** | **Sex** | **Age** | **Pathology** | **Location** | **Stage** | **Differentiation** |
| --- | --- | --- | --- | --- | --- | --- |
| 1 | M | 57 | NM | Asc | - | - |
| 2 | F | 50 | NM | Asc | - | - |
| 3 | F | 68 | NM | R | - | - |
| 4 | M | 59 | NM | Asc | - | - |
| 5 | M | 73 | NM | R | - | - |
| 6 | F | 54 | NM | Sc | - | - |
| 7 | F | 68 | NM | Sc | - | - |
| 8 | M | 80 | NM | Asc | - | - |
| 9 | M | 75 | TA | Sc | - | - |
| 10 | F | 64 | TA | R | - | - |
| 11 | F | 47 | TA | Tc | - | - |
| 12 | F | 68 | TA | RSc | - | - |
| 13 | M | 58 | TA | Dsc | - | - |
| 14 | M | 55 | TA | Asc | - | - |
| 15 | F | 63 | TA | R | - | - |
| 16 | M | 67 | TA | Hf | - | - |
| 17 | M | 47 | TVA | Dsc |  |  |
| 18 | F | 65 | TVA | Ce |  |  |
| 19 | M | 69 | TVA | R |  |  |
| 20 | M | 61 | TVA | Asc |  |  |
| 21 | F | 74 | TVA | Sc |  |  |
| 22 | F | 49 | TVA | Ce |  |  |
| 23 | F | 50 | TVA | RSc |  |  |
| 24 | M | 70 | TVA | Sc |  |  |
| 1 | M | 57 | AC | Ce | T3N0M0 | Moderate |
| 2 | F | 50 | AC | Ce | T3N0M0 | Moderate |
| 3 | F | 68 | AC | R | T2N0M0 | Well-Moderate |
| 4 | M | 59 | AC | Ce | T3N1bM0 | Well-Moderate |
| 5 | M | 73 | AC | R | T2N2aM0 | Moderate |
| 6 | F | 54 | AC | Sc | T3N0M0 | Moderate |
| 7 | F | 68 | AC | Sc | T3N2bM0 | Moderate |
| 8 | M | 80 | AC | Asc | T3N0M0 | Moderate |

Abbreviations: M, male; F, female; N, normal tissue; TA, tubular adenoma; TVA, tubulovillous adenoma; AC, adenocarcinoma; MAC, mucinous adenocarcinoma; Ce, Cecum; Asc, ascending colon; Hf, hepatic flexture; Tc, transverse colon; Dsc, descending colon; Sc, sigmoid colon; RSc, rectosigmoid colon; R, rectum.
