# Supplementary figures and images for: Exploration of the typical features of tubulovillous adenoma using in-depth quantitative proteomics analysis
Source: Bioengineered. 2021 Sep 29;12(1):6831–43. doi: 10.1080/21655979.2021.1971036 (PMC8806592; doi:10.1080/21655979.2021.1971036)

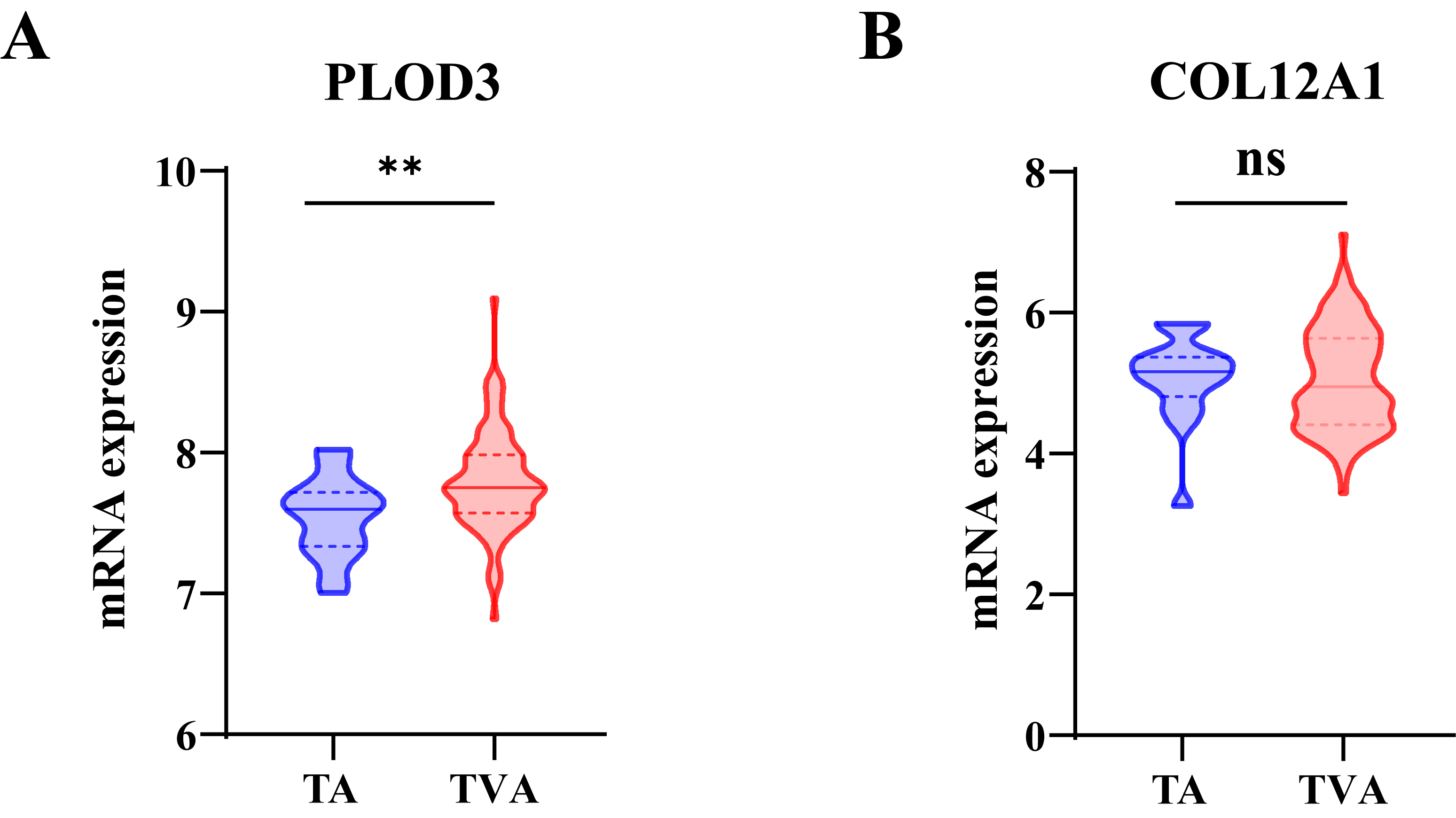

Supplement: Supplemental Material [file KBIE_A_1971036_SM7445.zip › supplementary/Figure S1.tif]

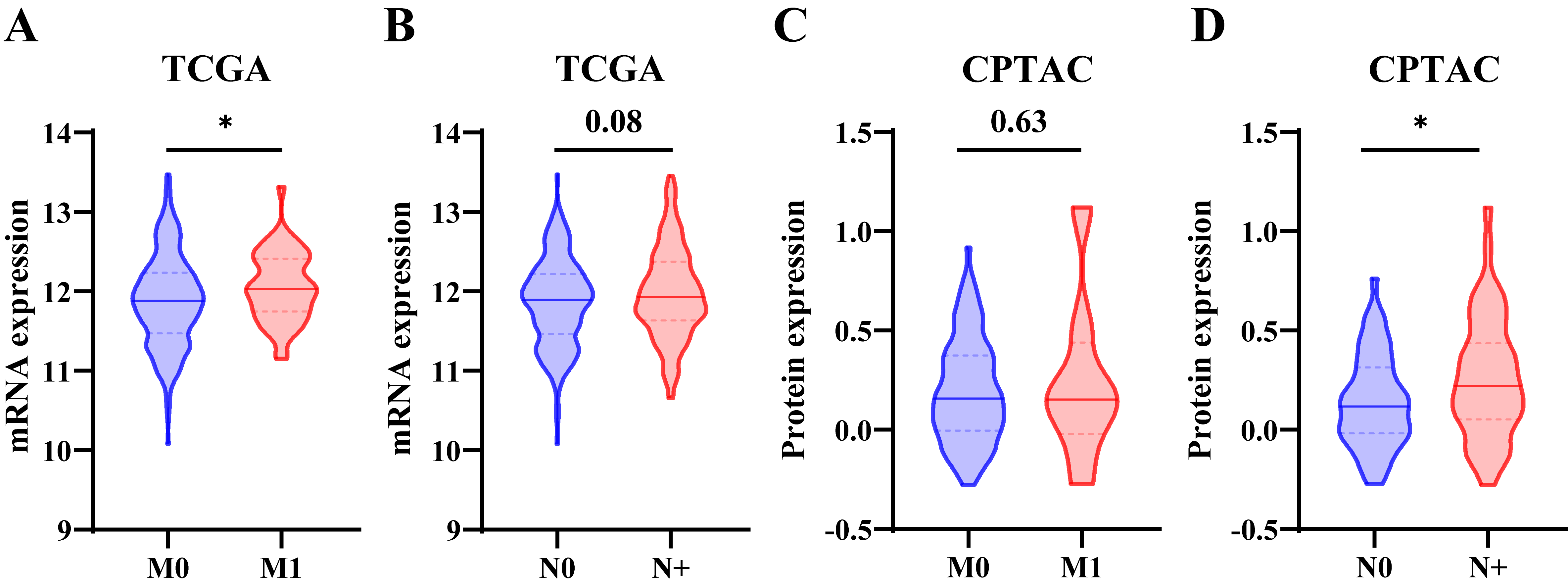

Supplement: Supplemental Material [file KBIE_A_1971036_SM7445.zip › supplementary/Figure S2.tif]

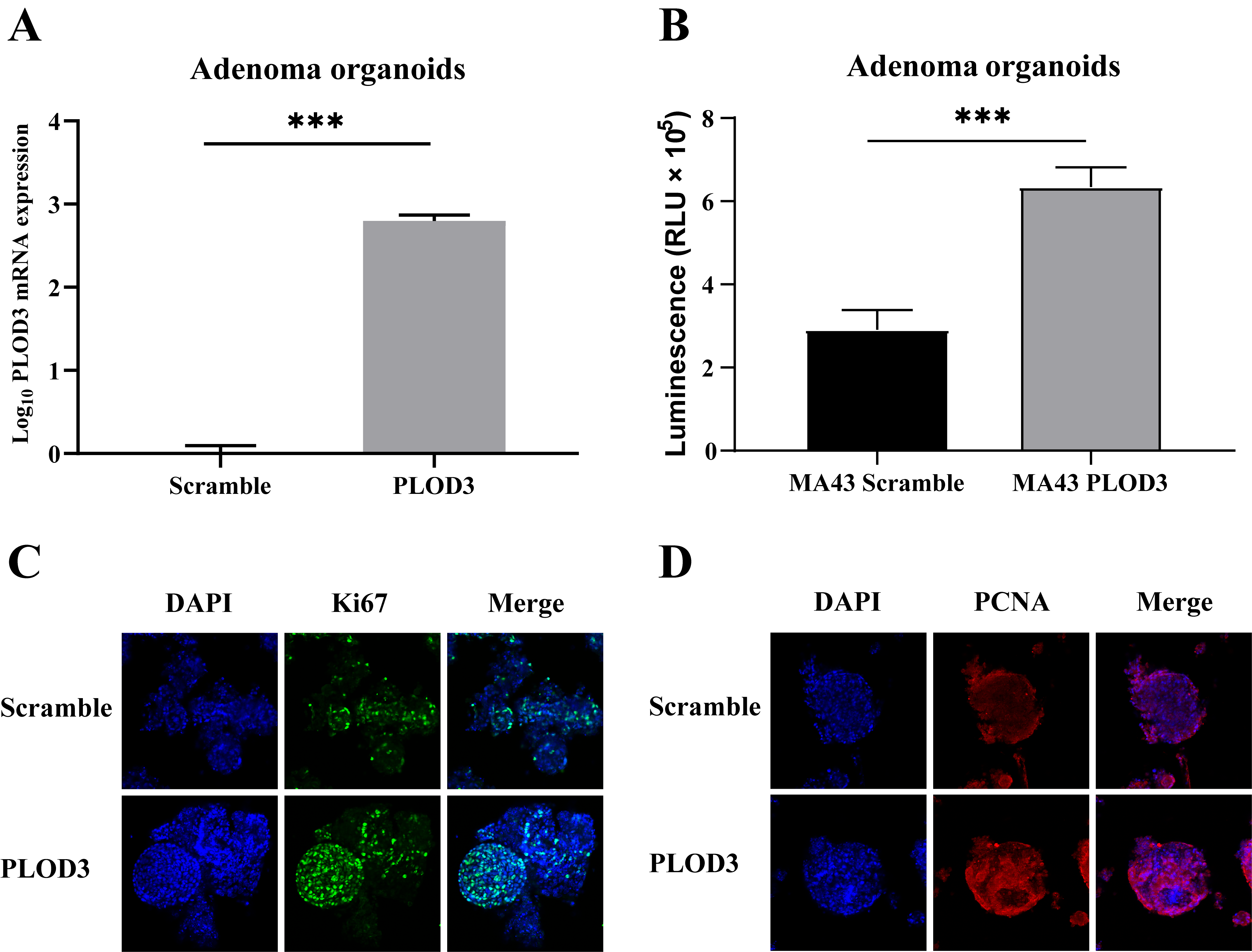

Supplement: Supplemental Material [file KBIE_A_1971036_SM7445.zip › supplementary/Figure S3.tif]
